# Comparison of bilateral eardrum temperatures measured using an infrared tympanic thermometer before and after surgery in patients with chronic otitis media

**DOI:** 10.1097/MD.0000000000030721

**Published:** 2022-10-28

**Authors:** Yee-Hyuk Kim

**Affiliations:** a Department of Otorhinolaryngology-Head & Neck Surgery, Daegu Catholic University School of Medicine, Daegu, Republic of Korea.

**Keywords:** ear canal, otitis media, surgery, temperature, thermometer, tympanic membrane

## Abstract

This study aimed to investigate the effect of chronic otitis media (COM) and COM surgery on infrared tympanic thermometer measurements. We retrospectively reviewed the medical records of 192 patients (192 surgery cases) who underwent surgery for COM and whose bilateral tympanic membrane temperature was measured with an infrared tympanic thermometer the day before surgery and at 2, 3, 4, and 6 months after surgery. Patients underwent surgery for COM in 1 ear, the other eardrum was intact. Patients who underwent tympanoplasty, simple mastoidectomy, and canal wall up mastoidectomy, surgeries performed to preserve the ear canal, were included in group A, and patients who underwent canal wall down mastoidectomy, a surgery to remove the ear canal, were included in group B. There were 115 and 77 patients in groups A and B, respectively. The mean temperature on the side with COM measured the day before surgery was 37.09°C ± 0.325°C and the mean temperature on the opposite normal side was 37.03°C ± 0.330°C (*P* = .000). In group A, the eardrum temperature on the surgical and contralateral side was not statistically different after surgery (*P* = .439). The temperature difference between both sides of the eardrums (dTemp) changed from 0.056°C before surgery to 0.014°C after surgery (*P* = .008). However, in group B, which canal wall down mastoidectomy was performed, the eardrum temperature of the surgical side was higher than that on the other side (*P* = .001). The dTemp increased up to 0.15°C after surgery (*P* = .000). The temperature of the eardrum was slightly increased by COM. The COM surgeries, which preserve the ear canal, brought the temperature of the eardrum close to that of the normal eardrum, and the surgery to remove the ear canal raised the temperature of the eardrum.

## 1. Introduction

Infrared tympanic thermometers (ITTs) are widely used in the medical field due to their distinct advantages, such as short measurement time, measurement convenience for both the user and the recipient, and accuracy.^[1–5]^ In addition, the frequency and the importance of measuring body temperature in daily life have increased during the COVID-19 pandemic. Till now, several research studies have assessed the effect of middle ear diseases, such as acute otitis media or otitis media with effusion, and minor ear surgery, such as ventilation tube insertion, on ITT measurements.^[6–13]^ However, there are very few studies evaluating the effect of chronic otitis media (COM) and surgery for COM on the measurement results of the ITT.^[13–15]^ Although there are studies showing that tympanic membrane perforation does not affect ITT measurements,^[13–15]^ these reports are only limited to central perforation of the eardrum and do not include various COM patients. Two papers on the effect of major ear surgery on ITT measurements reveal inconsistent findings. For instance, 1 report has shown that radical ear surgery lowers the temperature in children (0.53°C),^[13]^ whereas another report has demonstrated that canal wall down surgery increases the temperature in adults (0.66°C).^[15]^ This study aims to determine whether COM (chronic suppurative otitis media with tympanic membrane perforation, COM with cholesteatoma) and surgeries for COM affect the results of infrared tympanic thermometry in adults.

## 2. Materials and Methods

We retrospectively reviewed the medical records of 376 adults (386 surgery cases) aged 20 years or older who underwent surgery for COM between August 2015 and August 2020 at the Department of Otorhinolaryngology of Daegu Catholic University Medical Center in South Korea. Of them, we excluded 10 cases of surgical ear reoperation, 14 cases of COM in the contralateral ear, 9 cases of previous surgery for COM in the contralateral ear, and 161 cases that were not measured with an ITT either the day before the operation and at 2, 3, 4, and 6 months after surgery. Consequently, 192 subjects (192 surgeries) were included in this study. According to the abovementioned inclusion/exclusion criteria, 1 ear needed to have undergone COM surgery and the other ear needed to have a normal tympanic membrane. The types of surgeries for COM performed on the subjects were classified into tympanoplasty (TP), simple mastoidectomy (SM), canal wall up mastoidectomy (CWM), and canal wall down mastoidectomy (CWDM). The subjects who underwent TP, SM, and CWM, which are surgeries to preserve the external auditory canal, were referred to as group A, and those who underwent CWDM, which is a surgery to remove the external auditory canal, were referred to as group B.

First, we used paired *t* tests to identify the effect of COM on ITT results, by measuring the difference in eardrum temperatures between ears with a normal eardrum and ears with COM before surgery using an ITT.

Next, groups A and B were analyzed respectively by repeated measures ANOVA to determine whether there were differences in the bilateral eardrum temperature measured with an ITT at each postoperative period (on the 2nd, 3rd, 4th, and 6th month post-surgery). The purpose of this method was to investigate whether COM surgery affects the results of infrared tympanic thermometry.

Finally, by using the value obtained by subtracting the temperature of the tympanic membrane on the healthy control side from the tympanic membrane temperature on the operated side, the changing pattern of the temperature difference (dTemp) on both sides from before surgery to 6th month after surgery was analyzed by repeated measures ANOVA in groups A and B, respectively.

For statistical analysis, SPSS 25.0 (IBM Corp., Armonk, NY) was used, and a value of *P* < .05 was considered a statistically significant difference. This study was conducted with the approval of the Institutional Review Board of the Daegu Catholic University Medical Center (IRB No. CR-22-040).

## 3. Results

A total of 192 subjects (192 surgeries) were included in the study. The mean age was 55.4 ± 11.31 years (minimum: 22 years old, maximum: 79 years old), and there were 79 males and 113 females. Diagnostic diseases included 145 cases of chronic suppurative otitis media with tympanic membrane perforation and 47 cases of COM with cholesteatoma. There were 103 lesions on the right ear and 89 cases on the left ear. The operations performed were as follows: TP (n = 82), SM (n = 28), CWM (n = 5), and CWDM (n = 77), with 115 subjects in group A (TP, SM, and CWM) and 77 subjects in group B (CWDM) (Table 1).

**Table 1:**
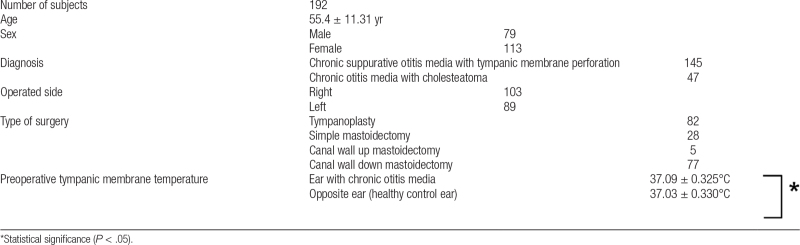
Summary of clinical data of patients and tympanic membrane temperature before surgery.

First, a comparison of the tympanic membrane temperatures of 192 subjects before surgery showed that the mean temperature of the eardrum was 37.09°C ± 0.325°C on the COM side and 37.03°C ± 0.330°C on the contralateral side, with a *P* value of .000. This result suggests that there was a statistically significant difference between the 2 measurements; thus, temperature of the lesion side with COM was higher than that of the healthy control side (Table 1).

**Figure 1.**
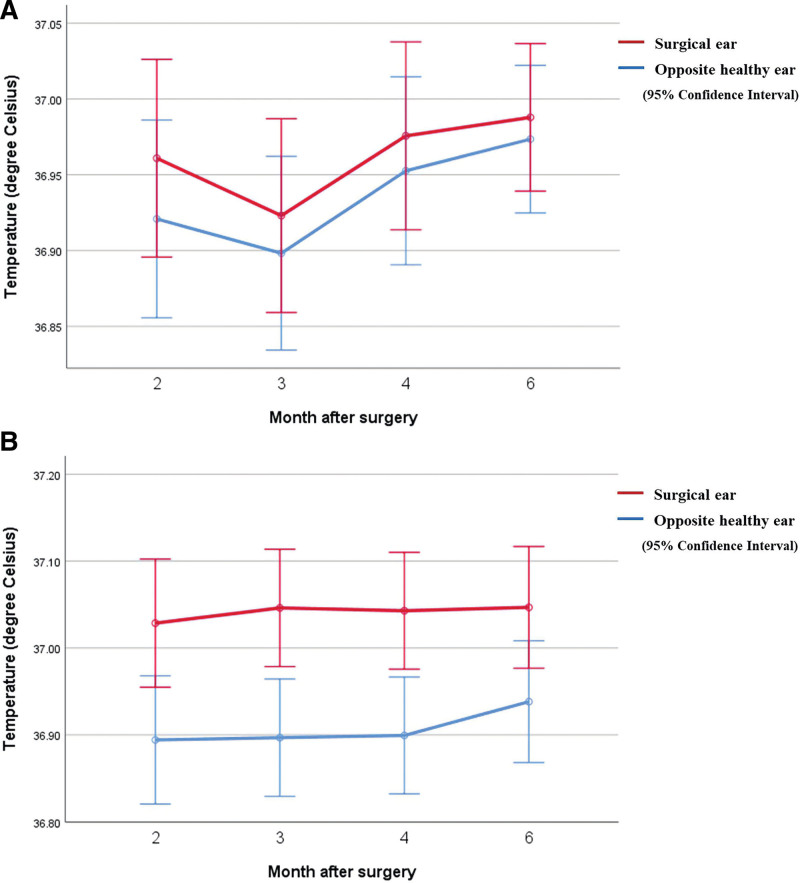
Comparison of bilateral tympanic membrane temperature after surgery. (A) Group A included patients who underwent tympanoplasty, simple mastoidectomy, and canal wall up mastoidectomy, in which the external auditory canal was preserved. Findings reveal no statistically significant difference between both eardrum temperatures after surgery (*P* = .439). (B) Group B included patients who underwent canal wall down mastoidectomy in which the external auditory canal was removed. Findings reveal that both eardrum temperatures show a statistically significant difference after surgery (*P* = .001).

Next, a comparison of the tympanic membrane temperatures on the surgical side and the contralateral eardrum after surgery revealed that there was no difference in the temperature of the tympanic membrane on both sides of group A (*P* = .439) (Fig. 1A). However, in group B, for which CWDM was performed, the tympanic membrane temperature on the surgical side was higher than that of the contralateral eardrum on the healthy control side after surgery, and there was a statistically significant difference between the tympanic membrane temperatures on both sides (*P* = .001) (Fig. 1B).

**Figure 2.**
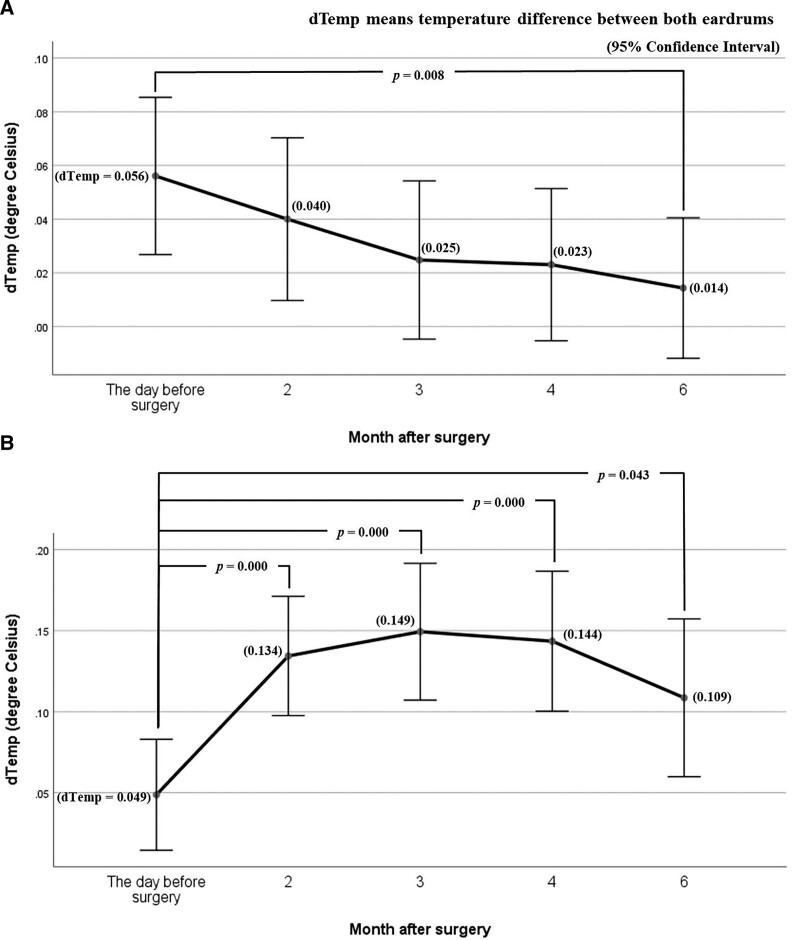
Changes in the temperature difference between both eardrums over time after surgery. (A) In group A, the difference in the temperature of both eardrums was the largest before surgery, and the difference gradually decreased after surgery, showing a statistically significant difference in the preoperative and postoperative 6th months (*P* = .008). It can be interpreted that chronic otitis media caused the difference in the temperature of the bilateral tympanic membranes before the operation. However, the inflammation in the middle ear disappeared, and the tympanic membrane was regenerated after the operation so that the difference in the temperature of both eardrums was reduced. (B) In group B, the difference in the temperature of both eardrums was greater after surgery than before surgery (*P* = .000 at 2, 3, and 4 months after surgery, *P* = .043 at 6 months postoperation) It can be interpreted that the ear canal was removed and meatoplasty was performed during surgery (canal wall down mastoidectomy), so the temperature of the tympanic membrane on the surgical side was statistically higher than the temperature measured in the normal tympanic membrane.

Finally, we examined the changing pattern of the difference in tympanic membrane temperature on both sides from before surgery to 6 months after surgery using dTemp. In group A, the difference in tympanic membrane temperature between both sides tended to gradually decrease over time after surgery. Compared with the preoperative value (0.056°C), the dTemp value at 6 months after surgery (0.014°C) was statistically significantly lower (*P* = .008) (Fig. 2A). However, in group B, the dTemp showed a statistically significant difference from the preoperative values in all periods between 2 and 6 months after surgery (*P* = .000 at 2, 3, and 4 months postoperation, *P* = .043 at 6 months postoperation). Furthermore, the dTemp in both eardrums was more prominent for all follow-up periods compared to before surgery. It revealed an increase of 0.11°C to 0.15°C until 6 months after surgery (Fig. 2B).

## 4. Discussion

When measuring the temperature of the eardrum in adults with an ITT, the auricle is pulled posteriorly and upward to make the cartilage portion of the external auditory canal as close to a straight line as possible between the sensor of the thermometer and the eardrum. In addition, when the probe of the ITT is inserted to a sufficient depth into the external auditory meatus, it helps straightening the cartilaginous external auditory canal. Consequently, the distance between the sensor of the thermometer and the tympanic membrane becomes closer and that the tympanic membrane temperature can be measured more accurately. However, if the probe of the ITT cannot be inserted deep enough because the entrance to the ear canal is not wide, the distance between the sensor of the thermometer and the eardrum increases, and the sensor and the eardrum are more likely to deviate from a straight line. In this case, the effect of eardrum radiation on the temperature measurement will decrease, and the influence of the external auditory canal radiation around the eardrum will relatively increase. Therefore, measurements may be substantially lower compared to the true value.^[16]^ When CWDM is performed, the bony portion of the external auditory canal is removed, and meatoplasty of the cartilaginous external auditory canal widens the entrance to the external auditory meatus, shortening the distance between the entrance of the external auditory meatus and the tympanic membrane. Since the entrance to the external auditory meatus is widened after CWDM, the probe of the ITT can be inserted deep enough, shortening the distance between the sensor of the thermometer and the tympanic membrane. Since the S-shaped external auditory canal is removed, the sensor of the thermometer and eardrum are more likely to be located in a straight line. For this reason, it can be considered that the temperature after surgery may be higher than before the operation when the temperature of the eardrum is measured with an ITT after CWDM.

In subjects who underwent TP, SM, and CWM surgery, the difference in bilateral tympanic membrane temperature was the largest before surgery, and the difference gradually decreased after surgery and was the smallest at 6 months after surgery. These findings suggest that the temperature of the eardrum increased due to COM before the operation. However, the inflammation in the middle ear cavity disappeared after the operation and the temperature of the normal eardrum on the opposite side of the operation was similar to that of the regenerated eardrum after the eardrum was regenerated.

The preoperative temperature can determine how COM affects the measurement value of the infrared tympanic thermometry. Several studies have reported that central tympanic perforation does not influence ITT measurements.^[13–15]^ Since the cause of central tympanic perforation in these reports is unclear, it is unsure whether patients with COM were included in these studies. In this study, the temperature of the eardrum with COM was higher than that of the normal eardrum, and the temperature difference between the bilateral eardrums was 0.06°C. Although this difference was statistically significant, it may be considered a minimal amount of change in clinical settings.

## 5. Conclusion

COM slightly increased the ITT measurements. The COM surgeries to preserve the external auditory canal brought the temperature of the eardrum closer to the normal eardrum than before the surgeries. The COM surgery to remove the external auditory canal significantly raised the temperature of the eardrum.

## Author contributions

Data curation: Yee-Hyuk Kim

Formal analysis: Yee-Hyuk Kim

Writing - original draft: Yee-Hyuk Kim

Writing - review & editing: Yee-Hyuk Kim
